# Cardioprotective effects of microRNA-18a on acute myocardial infarction by promoting cardiomyocyte autophagy and suppressing cellular senescence via brain derived neurotrophic factor

**DOI:** 10.1186/s13578-019-0297-8

**Published:** 2019-05-10

**Authors:** Bin Lin, Deguang Feng, Jing Xu

**Affiliations:** Department of Cardiovascular Surgery, The First Affiliated Hospital of Zhengzhou University, 1, Jianshe East Road, Zhengzhou, 450052 Henan People’s Republic of China

**Keywords:** MicroRNA-18a, Brain derived neurotrophic factor, Acute myocardial infarction, Akt/mTOR axis, Autophagy, Senescence

## Abstract

**Background:**

The prevention of cardiovascular diseases is a matter of great concern, of which acute myocardial infarction (AMI) remains one of the leading causes of death resulting in high morbidity worldwide. Emerging evidence highlights the importance of microRNAs (miRNAs) as functional regulators in cardiovascular disease. In this study, an AMI rat model was established in order to investigate the effect of miR-18a on cardiomyocyte autophagy and senescence in AMI and the underlying mechanism.

**Methods:**

In the present study, an AMI model was induced by ligating the anterior descending branch of left coronary artery in Wistar rats. Dual-luciferase reporter gene assay was introduced for exploration on the relationship between miR-18a and brain derived neurotrophic factor (BDNF). The gain- and loss-of-function experiments were performed to elucidate miR-18a and BDNF effects on cell autophagy and senescence in AMI by transfecting hypoxia-exposed H9c2 cells with miR-18a inhibitor or mimic, siRNA against BDNF, or hypoxia-exposed H9c2 cell treatment with an agonist of the Akt/mTOR axis (LM22B-10).

**Results:**

Upregulation of miR-18a was found in AMI, while downregulation was present in BDNF to activate the Akt/mTOR axis. Compared with the miR-18a inhibitor group, the expression of p-Akt and p-mTOR increased and the number of senescent cells increased in the miR-18a inhibitor + LM22B-10 group, and the expression of Beclin1, LC3-II, p62 decreased and autophagy decreased (all *p* < 0.05). Furthermore, this could be rescued by knocking down BDNF or Akt/mTOR axis activation by LM22B-10.

**Conclusion:**

All in all, downregulation of miR-18a could promote BDNF expression, which offers protection against AMI by inactivating the Akt/mTOR axis, highlighting a promising therapeutic strategy for AMI treatment.

## Background

As a major cause of death worldwide, acute myocardial infarction (AMI) is a consequence of acute coronary syndrome attributing to coronary artery obstruction such as coronary thrombosis [1, 2]. A variety of complications following AMI have been identified, including ventricular septal or papillary muscle rupture, apical left-ventricular thrombus formation, and pericardial effusion [3]. Approximately 17 million or more deaths for cardiovascular disease occur annually and the mortality rate for AMI patients accounts for 13% [4]. Based on the statistics, it is predicted that there will be 16 million people in 2020 and 7 million more in 2030 suffering from AMI in China, posing great risks [5]. In recent years, however, the underlying cellular mechanisms of cardiovascular disorders have been studied extensively, of which microRNAs (miRNAs) have attracted much research interests [6].

miRNAs, a group of small non-coding RNAs, have been reported to extensively play a pivotal role in multiple cellular biological processes such as cell apoptosis and proliferation, particularly in the cardiovascular system where miRNA regulate physiological and pathological events [7]. Moreover, several miRNAs, such as miR-451, miR-203, and miR-34, exert suppressive functions over cancer stemness and drug resistance in non-small cell lung cancer, breast cancer, and prostate cancer [8–10]. Recently, a number of miRNAs have been identified as potential diagnostic biomarkers and therapeutic targets for AMI, including miR-497, miR-19a, and miR-499 [11–13]. Besides, the miR-17-92 family and its paralogs have been reported to be involved in the process of angiogenesis following AMI, or other dyslipidemia and related diseases [13]. miR-18a, belonging to the miR-17-92 family, has emerged as a novel treatment for brain vascular diseases as a regulator of angiogenesis [14, 15]. However, whether miR-18a is involved in fine-tuning the function of cardiomyocytes requires further investigations. It is suggested that miR-18a regulates the brain derived neurotrophic factor (BDNF) and is able to modulate neuron plasticity and development, which is a pharmaceutical target in treating neural development and psychiatry disorders [16]. Considering the association with coronary artery disease, BDNF has also been reported as a therapeutic candidate for MI by ameliorating cardiac ischemic injury and suppressing cardiomyocyte apoptosis [17]. Additionally, the Akt/mammalian target of rapamycin (mTOR) axis has been reported to be involved in miR-28 regulated cardiomyocyte apoptosis in a previous study [18]. In the present study, the goal is to investigate miR-18a function in AMI in a rat model and the underlying mechanisms. It has been found that inhibition of miR-18a could upregulate BDNF, offering protection against AMI by inactivating the Akt/mTOR axis. Expectedly, the present study may provide a scientific rationale for the modulation of miRNAs in the treatment of cardiovascular disease.

## Materials and methods

### Animal treatment

Animal use and experimental procedures in this study were approved by the Experimental Animal Ethics Committee of The First Affiliated Hospital of Zhengzhou University. All operating procedures of animal experimental are in line with the United States National Institutes of Health (NIH) laboratory animal care and usage guidelines.

A total of 54 specific pathogen free (SPF) Wistar rats (aged 8–12 weeks; weighing 180–220 g; mean weight: [204 ± 11] g) were purchased from Shanghai Slac Laboratory Animal Co., Ltd. (Shanghai, China). The rats were housed in an atmosphere with 60% humidity at 25 °C with unlimited food and water supply and kept on a 12-h light/12-h dark cycle. The rats were anaesthetized using 3% pentobarbital sodium (P3761, Sigma-Aldrich Chemical Company, St. Louis, MO, USA) by intraperitoneal injection and monitored by a lead II electrocardiogram (ECG). Then, 16 rats were treated with sham-operation (the chest cavity of rats was opened and then statured), which served as a control. The remaining 38 rats were used for AMI rat model establishment, as described previously [19]. Briefly, the MI was induced by ligating the left anterior descending coronary arteries (LAD) at 2 mm below the left atrial appendage (LAA) of rats. Regional ischemia was confirmed by visual inspection for pallor or cyanosis. The AMI model was considered to be successfully established if lead ST segment elevation lasts for at least 15 min. Moreover, the rats were intraperitoneally injected with 5 mL of normal saline and intramuscularly injected with 0.5 mL of 80,000 U penicillin sodium after model establishment as a precaution against infections. The successful rate of AMI model establishment was 84.00% (32/38). Subsequently, the 32 AMI rats were randomly distributed in two groups and via the tail vein injected with miR-18a antagonist (5 mg/kg; RiboBio Co., Ltd., Guangzhou, Guangdong, China) or negative control (NC) plasmids 24 h after modeling, respectively (n = 16 for each group).

The rats were anaesthetized by intraperitoneal injection of 3% pentobarbital sodium (P3761, Sigma-Aldrich Chemical Company, St. Louis, MO, USA) at 48-h post plasmid injections and the hearts were obtained. The rats were then subjected to the 2,3,5-triphenyltetrazolium chloride (TTC) staining (n = 9 in each group), enzyme linked immunosorbent assay (ELISA) (n = 4 in each group), immunohistochemistry, reverse transcription quantitative polymerase chain reaction (RT-qPCR), and Western blot analysis (in the last three experiments, n = 3 in each group).

### Myocardial infarct size (IS) assessment

The obtained rat hearts were stored at − 80 °C for 20 min and further sliced into 5 sections. The sections were incubated in 1% TTC solution for 15 min at 37 °C in void of light. Furthermore, the size of infarct area (IS, stained as gray) and non-infarct area (NIS, stained as red) were quantified using Image J and the ratio of IS/myocardium size (MS) was calculated.

### Measurement of BDNF level

ELISA was performed in order to determine BDNF expression in rat heart. The hearts were rinsed in normal saline to remove excess blood and homogenized mechanically. Moreover, the tissue was centrifuged (4 °C, 5000 rpm, 10 min) and the supernatant was collected. The standard sample was diluted and added into the blank well, while the supernatant of corresponding groups was added into other wells, and incubated at 37 °C for 90 min. The blank well was added using 1% bovine serum albumin (BSA) after being washed 5 times with 0.15 M PBS. Other wells were added with the biotinylated antibody working solution, sealed, and incubated at 37 °C for 60 min. The plate was then sealed and incubated at 37 °C for an additional 30 min after the enzyme conjugate diluent and the enzyme conjugate were added. Finally, the termination solution was added following the chromogenic substrate addition, sealing, and incubation. BDNF content was determined using an ELISA kit (Promega Corp., Madison, WI, USA) in accordance to the manufacturer’s instructions. The optical density (OD) values were measured using a microplate reader (BioTek Instruments, Winooski, VT, USA) at the wavelength of 450 nm.

### Immunohistochemistry

The IS of the rat was cut off, fixed using 10% formaldehyde for 24 h, embedded with paraffin, and further sliced. The slices were dewaxed by applying xylene and dehydrated by different concentrations of alcohol (100% I, 100% II, 95%, 85%, and 75% successively). All slices were then immune-stained overnight with rabbit anti-BDNF (1:500, ab205067, Abcam Inc., Cambridge, MA, USA) at 4 °C, and incubated with goat anti-rabbit secondary antibody (ab6721, 1:1000, Abcam Inc., Cambridge, MA, USA) at room temperature for 60 min. Following a phosphate buffer saline (PBS) wash, the slices were further incubated with horseradish peroxidase (HRP)-labeled streptavidin-peroxidase at room temperature for 20 min and developed with diaminobenzidine (DAB).

### Dual-luciferase reporter gene assay

BDNF and miR-18a sequences were retrieved from National Center for Biotechnology Information (NCBI) database (http://www.ncbi.nlm.nih.gov/gene). The wild type (wt)-3′-untranslated region (UTR) of BDNF and mutant (mut)-3′-UTR of BDNF in which the potential miR-18a binding sites were mutated were cloned into pisCHECK2 vector (Ambion, Thermo Fisher Scientific Inc., San Jose, CA, USA). NC or miR-18a mimic was transfected into 293T cells with either BDNF-wt or BDNF-mut luciferase reporter plasmids. The dual-luciferase reporter assay kit (Promega Corp., Madison, WI, USA) was employed to determine luciferase activity in cell lysis using a luminescence detector (GloMax2020, Promega Corp., Madison, WI, USA) [20].

### Cell treatment

Rat cardiomyocytes H9c2 cells were purchased from American Tissue Culture Collection (ATCC, Rockville, MD, USA). H9c2 cells were cultured in a Dulbecco’s Modified Eagle Medium (DMEM; Gibco, Grand Island, NY, USA) containing 10% fetal bovine serum (FBS; Gibco, Grand Island, NY, USA), 10 mM 2-[4-(2-Hydroxyethyl)-1-piperazinyl] ethanesulfonic acid (HEPES; Sigma-Aldrich Chemical Company, St Louis, MO, USA), and 100 U/mL penicillin–streptomycin in an incubator (Thermo Fisher Scientific Inc., San Jose, CA, USA) with 5% CO_2_ at 37 °C. The cells were then exposed to hypoxia condition in a water-jacketed CO_2_ incubator (Series 8000WJ, Thermo Fisher Scientific Inc., San Jose, CA, USA) at 37 °C with 94% N_2_, 5% CO_2_, and 1% O_2_ for a total of 12 h.

The H9c2 cells at the logarithmic growth phase were seeded into a 12-well plate with 10^6^ cells/mL. After a 12-h starvation with serum-free medium, the cells were transfected with the following plasmids, respectively: siRNA against BDNF, miR-18a mimic (50 nM), miR-18a inhibitor (100 nM), agonist of the Akt/mTOR axis (LM22B-10), and negative control (NC) using lipo2000. All the aforementioned plasmids were provided by RiboBio Co., Ltd. (Guangzhou, Guangdong, China). After an additional 6 h of culture, the medium was replaced with DMEM complete medium.

### RNA isolation and quantification

Total RNA in the tissues and H9c2 cells was extracted using Trizol (Invitrogen Inc., Carlsbad, CA, USA). The cDNA was reversely transcribed using oligodT method. The qPCR was carried out using SYBR green Master kit to determine target gene expression. The primers were synthesized by Beijing Genomics Institute (Beijing, China) (Table 1). U6 or GAPDH was used as an internal control and 2^−ΔΔCt^ method was implemented to calculate the fold changes. Each individual experiment was repeated 3 times.

**Table 1:** Primer sequences for reverse transcription quantitative polymerase chain reaction

Gene	Primer sequences (5′–3′)
miR-18a	F: GCTGAGCTAAGGTGCATCTAG
	R: TCAACTGGTGTCGTGGAGT
BDNF	F: GCGCCCATGAAAGAAGCA
	R: CACAGCTGGGTAGGCCAAGT
U6	F: TGCGGGTGCTCGCTTCGGCAGC
	R: CCAGTGCAGGGTCCGAGGT
GAPDH	F: GCAAGTTCAACGGCACAG
	R: ACGCCAGTAGACTCCACGAC

miR, microRNA; BDNF, brain derived neurotrophic factor; GAPDH, glyceraldehyde-3-phosphate dehydrogenase; F, forward, R, reverse

### Western blot analysis

The tissues and H9c2 cells were lysed by radio immunoprecipitation assay (RIPA) lysis buffer (Beyotime Biotechnology Co., Ltd., Shanghai, China) and centrifuged (14,000 rpm, 4 °C). The supernatant was collected to obtain the total protein. The concentration of the extracted protein was determined using the bicinchoninic acid (BCA; Pierce Chemical, Dallas, Texas, USA). Furthermore, 4% concentration gel and 10% separation gel were used to separate the protein, which was then transferred to a membrane. The membrane was blocked with 5% BSA and incubated with the following primary antibodies at a dilution of 1: 5000: mouse monoclonal to BDNF (ab205067), rabbit polyclonal to p-Akt (ab38449), rabbit monoclonal to Akt (ab179463), rabbit polyclonal to mTOR (ab2732), rabbit monoclonal to p-mTOR (ab137133), rabbit monoclonal to Beclin1 (ab207612), rabbit polyclonal to light chain 3 (LC3)-II/I (ab128025), rabbit polyclonal to P62 (ab91526), and rabbit polyclonal to GAPDH (ab9485) at 4 °C overnight. All the antibodies were purchased from Abcam Inc. (Cambridge, MA, USA). Moreover, the membrane was incubated with the secondary antibody, HRP-conjugated goat anti-rabbit antibody to IgG (1:5000, ab205718) at room temperature for 2 h. Finally, the enhanced chemiluminescence (ECL) reagent (Invitrogen Inc., Carlsbad, CA, USA) was applied to visualize the bands, which were further analyzed by a Bio-rad Microscopic Imaging System and the Image software. Each individual experiment was repeated 3 times.

### Immunofluorescence staining

The rat cardiomyocytes were seeded in a 24-well plate and infected with AAV-GFP-LC3 (GeneChem Co., Ltd., Shanghai, China) (multiplicity of infection = 1:25). Following incubation at room temperature for a total of 2 h, the culture medium was replaced with fresh complete medium and cardiomyocytes were cultured for an additional 48 h in an incubator at 37 °C with 5% CO_2_. Furthermore, the cells were fixed, mounted, and observed under a fluorescence microscope (Nikon Corp., Tokyo, Japan) with the number of autophagosomes totaled (green fluorescence) [21].

### Senescence-associated β-galactosidase (SA-β-gal) staining

The rat cardiomyocytes were seeded into a 6-well plate and subjected to SA-β-gal staining as previously described [22]. The cytoplasm of senescent cells was blue-stained, regarded as positive cells. Photos were taken and observed under the inverted microscope (200×) with 3 fields of view randomly selected from each well. The proportion of positive cells was then calculated and 5 duplicated wells were set in individual groups.

### Superoxide dismutase (SOD) and malondialdehyde (MDA) measurement

SOD and MDA contents in H9c2 cells were measured by ELISA kit (Promega Corp., Madison, WI, USA) in accordance to the manufacturer’s instructions. The absorbance was measured at the wavelength of 450 nm using a microplate reader (BioTek Instruments, Winooski, VT, USA). The standard curve was then plotted, from which the contents of SOD and MDA were calculated [23].

### Statistical analysis

All data were processed using SPSS 21.0 statistical software (IBM Corp. Armonk, NY, USA). The measurement data were expressed by mean ± standard deviation. The normal distribution and variance homogeneity were initially measured. As for data fitting normal distribution and variance homogeneity, non-paired *t*-test was employed for comparison between two groups, one-way analysis of variance (ANOVA) or repeated measurement ANOVA was used for comparisons among multiple groups, and pairwise comparison within one group was tested by post hoc. *p* < 0.05 was indicative of significant statistical difference.

## Results

### miR-18a is highly expressed and BDNF is poorly expressed in a rat model of AMI

**Fig. 1:**
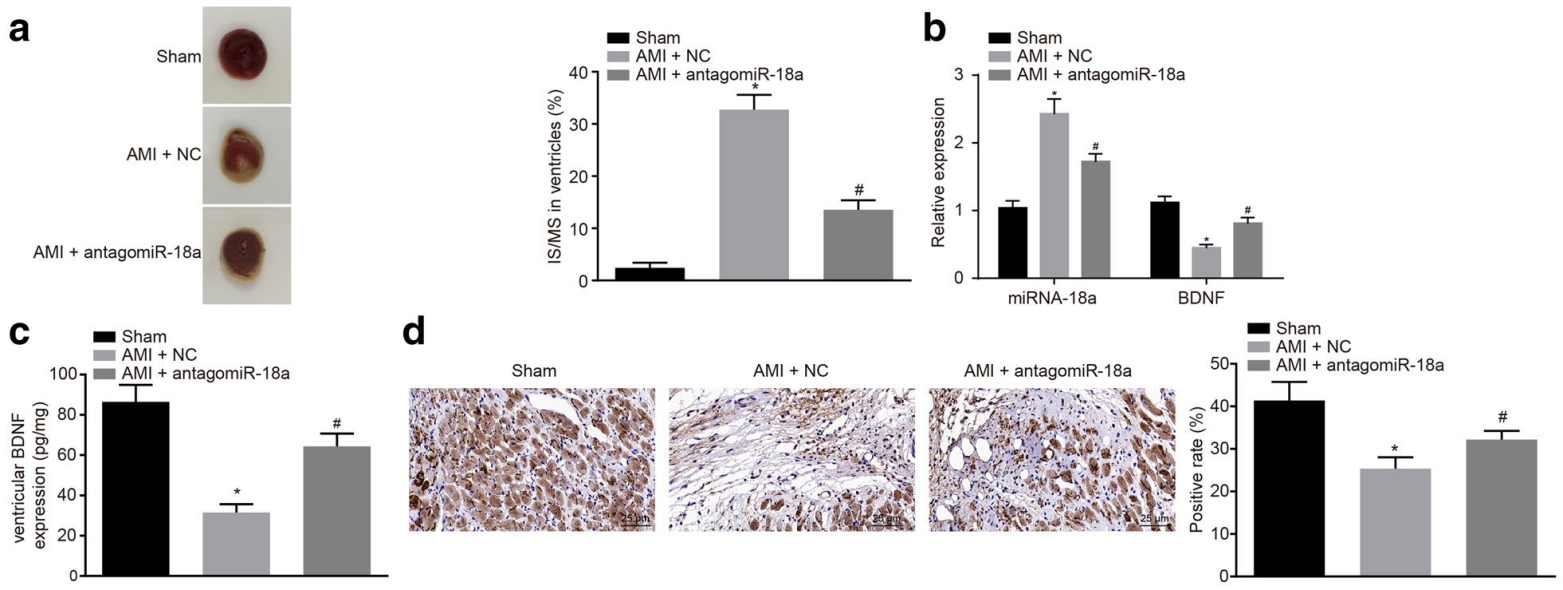
miR-18a is highly expressed and BDNF is poorly expressed in rats with AMI. **a** IS/MS determined by TTC staining (n = 9); **b** miR-18a and BDNF expression determined by RT-qPCR (n = 3); **c** ventricular BDNF expression determined by ELISA (n = 4); **d** BDNF expression in IS determined by immunohistochemistry (n = 3). The data were measurement data and expressed as mean ± standard deviation. The one-way analysis of variance was employed for data analysis among multiple groups; *p < 0.05 vs. the sham group; ^#^*p* < 0.05 vs. the AMI + NC group

Initially, the AMI rat model was successfully established and both miR-18a and BDNF expression was determined using RT-qPCR and ELISA. Furthermore, TTC staining was performed to identify IS and NIS. It was evident that there was an increase of IS/MS in the AMI + NC group in comparison to the sham group, while IS/MS decreased in the AMI + antagomiR-18a group in comparison to the AMI + NC group (*p* < 0.05, Fig. 1a). Simultaneously, RT-qPCR results displayed an increased miR-18 expression where BDNF was expressed at a lower level in IS in the AMI + NC group. Comparatively, these results were relatively higher than those in the sham group. Opposite results were found in the AMI + antagomiR-18a group in comparison to the AMI + NC group (*p* < 0.05, Fig. 1b–d). On the other hand, there was no significant difference in miR-18a expression in the surrounding area among the 3 groups (*p* > 0.05). These results confirm the fact that miR-18a was highly expressed while BDNF was poorly expressed in rats with AMI.

### miR-18a targets and reduces BDNF

**Fig. 2:**
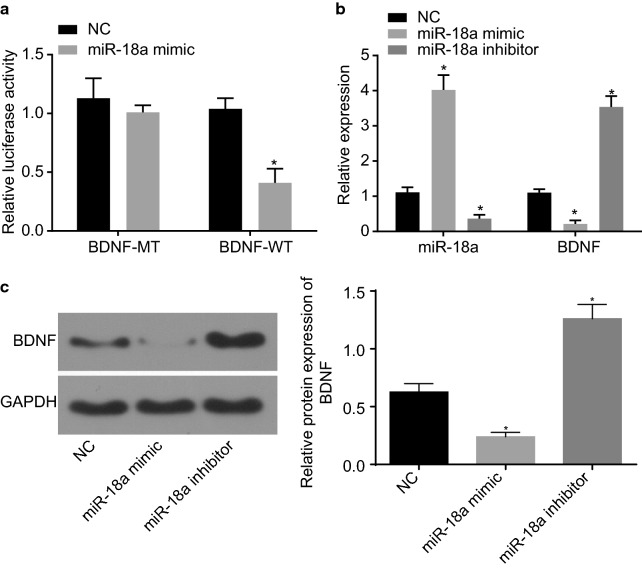
BDNF is confirmed to be a target gene of miR-18a. **a** Dual-luciferase reporter assay for confirmation of the relationship between miR-18a and BDNF; **b** the miR-18a expression and mRNA expression of BDNF in H9c2 cells assessed by RT-qPCR; **c** the protein expression of BDNF in H9c2 cells assessed by western blot analysis. The data were measurement data and expressed as mean ± standard error. Comparison was conducted by non-paired *t*-test between two groups and by one-way analysis of variance among multiple groups. Each experiment was repeated 3 times; *p < 0.05 vs. the NC group

Subsequently, dual-luciferase reporter gene assay was conducted to verify whether a targeting relationship exists between miR-18a and BDNF. As shown in Fig. 2, when compared with the BDNF-wt + NC group, the luciferase activity of BDNF-wt was deteriorated in the BDNF-wt + miR-18a mimic group (*p* < 0.05, Fig. 2a). When compared with the NC group, miR-18a delivered a higher expression while BDNF withheld a lower expression. These results were found in the miR-18a mimic group while the miR-18a inhibitor group exhibited paradoxical results (*p* < 0.05, Fig. 2b, c). This statistical evidence indicates that the miR-18a targeted and negatively regulated BDNF.

### Down-regulated miR-18a promotes the hypoxia-induced H9c2 cell autophagy by up-regulating BDNF expression

**Fig. 3:**
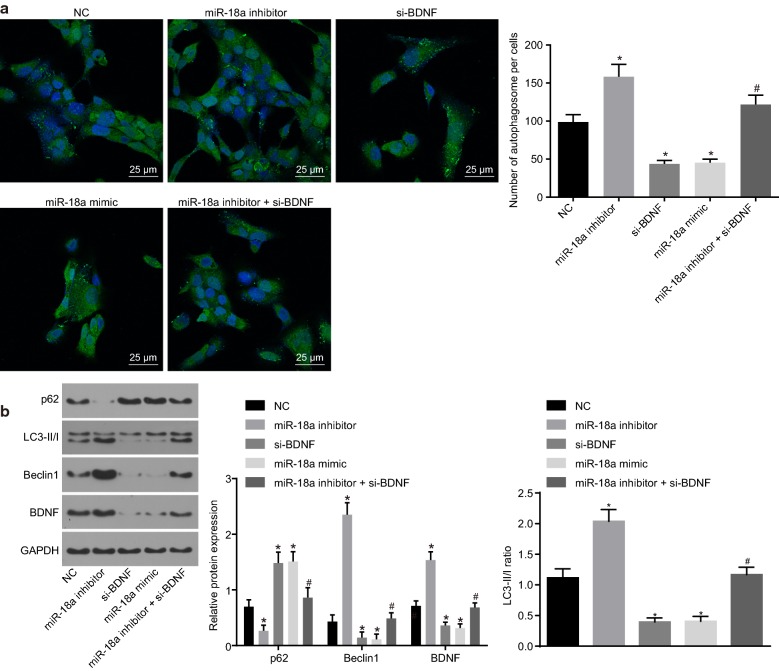
Down-regulated miR-18a promotes the hypoxia-induced H9c2 cell autophagy by promoting BDNF expression. **a** The number of autophagosomes induced by hypoxia quantified by immunofluorescence (×400); **b** the protein expression of BDNF and autophagy-related proteins (Beclin1, LC3-II/I and p62) determined by western blot analysis. The data were measurement data and expressed as mean ± standard error. Comparison was conducted by one-way analysis of variance among multiple groups. Each experiment was repeated 3 times; *p < 0.05 vs. the NC group; ^#^*p* < 0.05 vs. the miR-18a inhibitor group

Then, immunofluorescence was introduced to identify and distinguish the effects of miR-18a expression and BDNF on the autophagy-related markers (Beclin1, LC3-II/I and p62) of the H9c2 cells. Furthermore, after elaborately evaluating the effects upon the expressions, it was observed that there were more autophagosomes in the miR-18a inhibitor group and fewer in the si-BDNF and miR-18a mimic groups compared with the NC group. It was proven that as a matter of course, the amount of autophagosomes undoubtedly reduced in the miR-18a inhibitor + si-BDNF group in comparison with that in the miR-18a inhibitor group (*p* < 0.05, Fig. 3a). In contrast to the NC group, the expression of BDNF, Beclin1, and LC3-II/I increased, while expression of p62 decreased in the miR-18a inhibitor group. Although, the si-BDNF and miR-18a mimic groups displayed a coherent decrease in the expression of BDNF, Beclin1, and LC3-II/I, whereas an increase in expression of p62. Moreover, a more subordinate expression of BDNF, Beclin1 and LC3-II/I and a higher expression of p62 were evaluated in the miR-18a inhibitor + si-BDNF group than those found in the miR-18a inhibitor group (*p* < 0.05, Fig. 3b). These findings suggested that hypoxia-induced autophagy of H9c2 cells were heightened by inhibiting miR-18a through upregulating BDNF.

### Down-regulated miR-18a inhibits hypoxia-induced H9c2 cell senescence by up-regulating BDNF expression

**Fig. 4:**
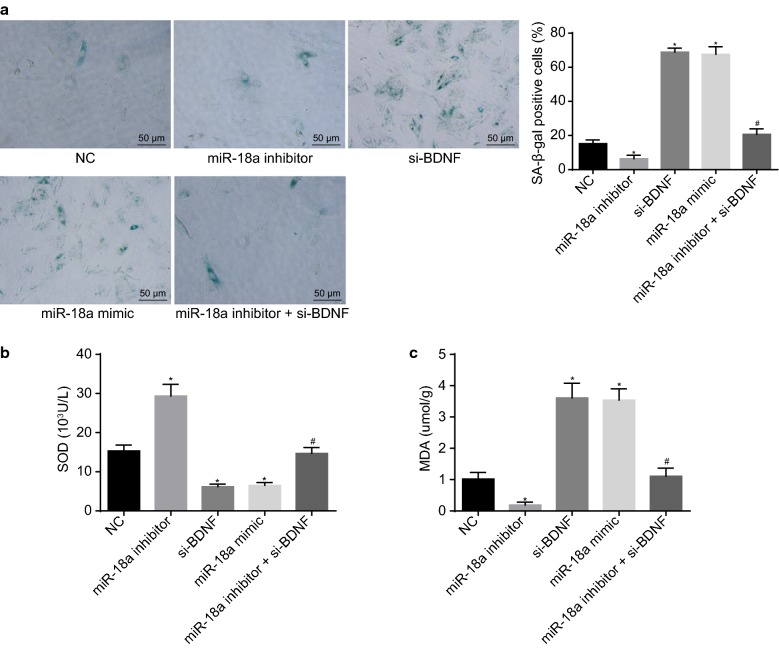
Repressed miR-18a inhibits the hypoxia-induced H9c2 cell senescence by up-regulating BDNF expression. **a** The H9c2 cell senescence induced by hypoxia identified by SA-β-gal staining (×200); **b**, **c** levels of SOD and MDA in H9c2 cells after exposure to hypoxia measured by ELISA. The data were measurement data and expressed as mean ± standard error. Comparison was conducted by one-way analysis of variance among multiple groups. Each experiment was repeated 3 times; *p < 0.05 vs. the NC group; ^#^*p* < 0.05 vs. the miR-18a inhibitor group

Next, the effects of miR-18a on the hypoxia-induced H9c2 cell senescence were thoroughly observed by SA-β-gal staining. As depicted in Fig. 4a, in comparison to the NC group, the rate of SA-β-gal positive cells reduced in the miR-18a inhibitor group. Comparatively, it was increased in the siRNA-BDNF and miR-18a mimic groups. Additional SA-β-gal positive cells were identified in the miR-18a inhibitor + siRNA-BDNF group than those in the miR-18a inhibitor group (*p* < 0.05). Besides, SOD and MDA levels were measured to evaluate cell senescence. An increased level of SOD and a decreased MDA level were evident in the miR-18a inhibitor group, while the level of SOD reduced and the level of MDA elevated in the si-BDNF and miR-18a mimic groups when compared to the NC group (*p* < 0.05). Lower SOD level and higher MDA levels were displayed in the miR-18a inhibitor + si-BDNF in comparison to the miR-18a inhibitor group (*p* < 0.05, Fig. 4b). The results elucidate that H9c2 cell senescence induced by hypoxia can be suppressed by down-regulation of miR-18a through up-regulation of BDNF.

### Down-regulation of miR-18a inactivates the Akt/mTOR axis by up-regulating BDNF expression

**Fig. 5:**
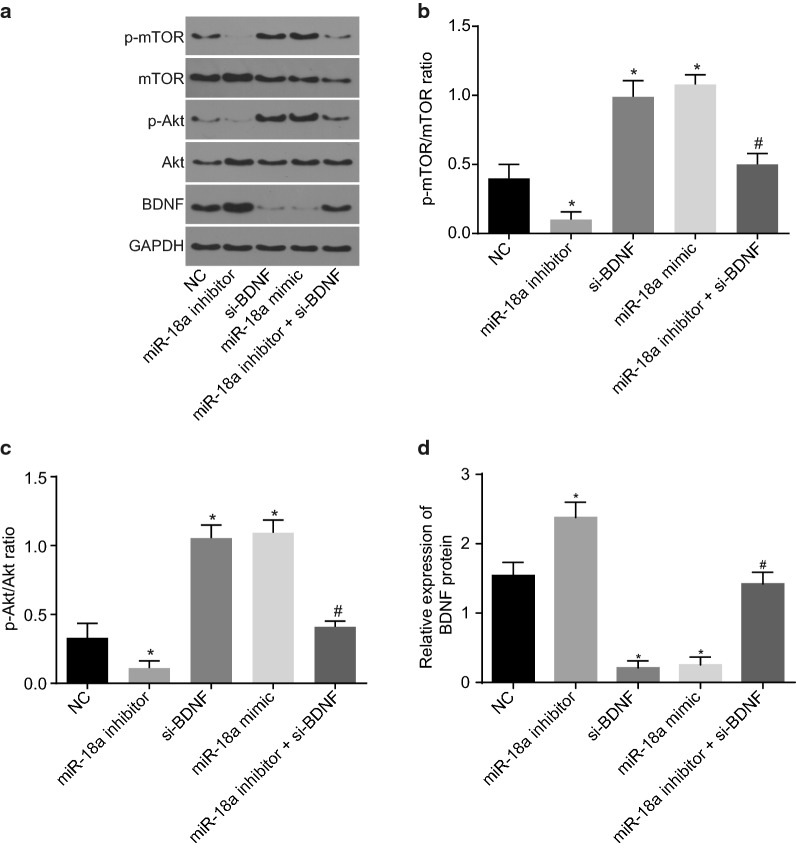
Inhibited miR-18a impedes the Akt/mTOR axis by up-regulating BDNF. **a** The gray value of p-Akt, Akt, p-mTOR, mTOR and BDNF protein bands in response to the treatment of miR-18a mimic, inhibitor and/or BDNF silencing; **b** the ratio of p-mTOR to mTOR in response to the treatment of miR-18a mimic, inhibitor and/or BDNF silencing; **c** the ratio of p-Akt to Akt in response to the treatment of miR-18a mimic, inhibitor and/or BDNF silencing; **d** the protein expression of BDNF in response to the treatment of miR-18a mimic, inhibitor and/or BDNF silencing evaluated by western blot analysis. The data were measurement data and expressed as mean ± standard error. Comparison was conducted by one-way analysis of variance among multiple groups. Each experiment was repeated 3 times; *p < 0.05 vs. the NC group; ^#^*p* < 0.05 vs. the miR-18a inhibitor group

In order to investigate whether an interaction was present among miR-18a, BDNF, and Akt/mTOR axis, the expression of the Akt/mTOR axis-related proteins was evaluated using a western blot analysis. The use of western blot analysis assisted in figuring out the regulation of Akt/mTOR axis by miR-18a. Figure 5 shows that in comparison to the NC group, miR-18a inhibitor increased BDNF expression, and lowered the extent of Akt and mTOR phosphorylation, while BDNF knockdown and miR-18a overexpression diminished the expression of BDNF, and increased the extent of Akt and mTOR phosphorylation. In contrary to miR-18a inhibitor alone, silencing BDNF could rescue the reduced extent of Akt and mTOR phosphorylation due to miR-18a inhibition (*p* < 0.05). It can be concluded that down-regulated miR-18a blocks the Akt/mTOR axis by up-regulating BDNF expression.

### Down-regulation of miR-18a promotes hypoxia-induced autophagy and inhibits hypoxia-induced senescence of H9c2 cells through inhibition of the Akt/mTOR axis

**Fig. 6:**
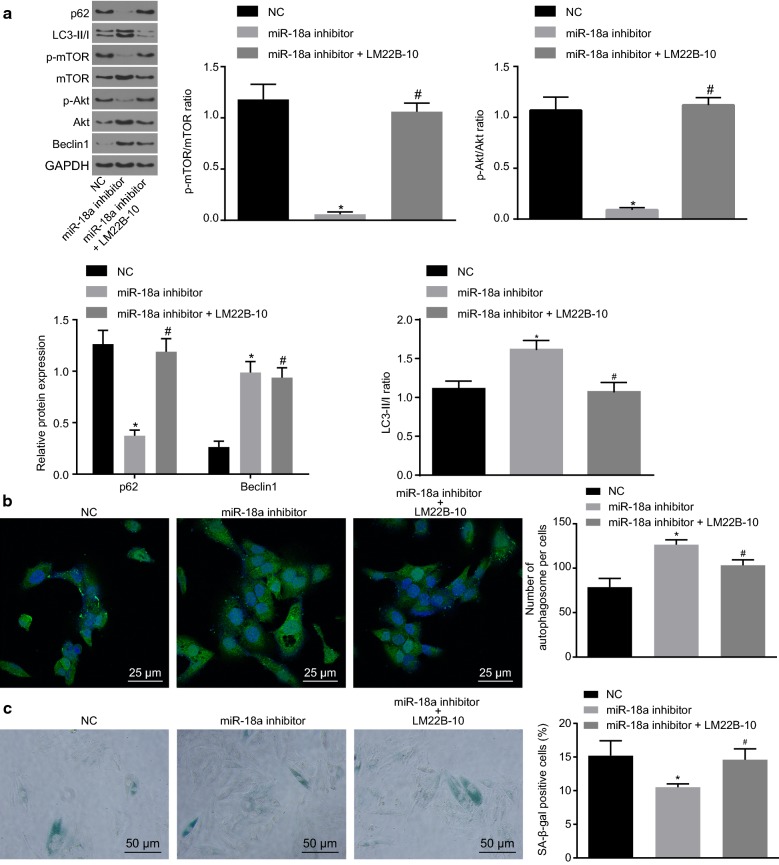
Down-regulating miR-18a promotes hypoxia-induced H9c2 cell autophagy and inhibits hypoxia-induced cellular senescence through suppressing the Akt/mTOR axis. **a** Protein expression of autophagy-related markers (Beclin1, LC3-II/I and p62) in H9c2 cells determined by western blot analysis; **b** the number of autophagosomes in H9c2 cells after exposure to hypoxia identified by GFP-LC3 (×400); **c** the H9c2 cell senescence after exposure to hypoxia identified by SA-β-gal staining (×200). The data were measurement data and expressed as mean ± standard error. Comparison was conducted by one-way analysis of variance among multiple groups. Each experiment was repeated 3 times; *p < 0.05 vs. the NC group; ^#^*p* < 0.05 vs. the miR-18a inhibitor group

Lastly, the involvement of the Akt/mTOR axis in the regulation of hypoxia-induced autophagy and senescence of H9c2 cells by miR-18a was observed. Both Beclin1 and LC3-II/I expression was elevated, while the extent of Akt and mTOR phosphorylation as well as the expression of p62 was repressed by miR-18a inhibitor. The trends could be reversed by LM22B-10, an agonist of the Akt/mTOR axis (*p* < 0.05, Fig. 6a). Furthermore, more autophagosomes were detected in the miR-18a inhibitor group than in the NC group, which were diminished in response to LM22B-10 treatment (*p* < 0.05, Fig. 6b). SA-β-gal staining revealed fewer senescent cardiomyocytes found in the miR-18a inhibitor group than in the NC group. In contrary, senescent cardiomyocytes were found in the miR-18a inhibitor + LM22B-10 group than in the miR-18a inhibitor group (*p* < 0.05, Fig. 6c). The aforementioned findings indicated that down-regulated miR-18a could promote hypoxia-induced autophagy and inhibit hypoxia-induced senescence of H9c2 cells through suppression of the Akt/mTOR axis.

## Discussion

AMI is able to induce sudden cardiac death, leading to numerous cases of death and disability worldwide [24]. Emerging evidence has highlighted the regulatory role of miRNAs in cardiac pathophysiology. For example, miR-22 down-regulation has been revealed to activate cardiac autophagy, which can contribute to improvement of cardiac function following MI [25]. With the purpose to uncover the role of miR-18a in AMI, the present study has demonstrated that down-regulation of miR-18a can promote autophagy and inhibit senescence of H9c2 cells induced by hypoxia by upregulating BDNF and inactivating the Akt/mTOR axis, playing an important role in protecting the cells from AMI.

Initially, it was discovered that miR-18a was highly expressed in cardiomyocytes of rats with AMI, while, in contrary, BDNF exhibited a low expression level. Consistent with the findings, miR-133, miR-663b, and miR-1291 have all been found to be highly expressed in AMI according to a previous study [26]. It is worth noting that higher levels of miR-208b and miR-34a have been demonstrated to reflect higher risk of death or heart failure in left ventricular remodeling following AMI [27]. Decreased BDNF expression has also been validated as a consequence of cardiac dysfunction induced by doxorubicin [28]. Besides, 3′UTR of BDNF contains conserved binding sites for various miRNAs, of which miR-1, miR-10b, miR-155, and miR-191 have been displayed to regulate BDNF expression [29]. These findings also confirmed that BDNF is a target gene of miR-18a by luciferase reporter assay.

Additionally, down-regulation of miR-18a was proven to exert encouraging effects on cell autophagy by promoting BDNF expression and inhibiting the Akt/mTOR axis, which was reflected by higher levels of Beclin1, LC3-II/I, and lower levels of p62. Similar to the results of the present study, miR-21 has been indicated to suppress autophagy of H9c2 cells via Akt/mTOR axis in cardiac hypoxia/reoxygenation-induced injury [30]. Autophagy is an evolutionarily conserved pathway within cells to degrade unnecessary or dysfunctional components, and to maintain autophagy is essential in cardiac homeostasis control under certain stress conditions [31]. Beclin1, a key molecule in the autophagic machinery that can be regulated by miRNAs, has been suggested to regulate autophagy in the heart [32]. Besides, the ratio of LC3-II to LC3-I has been found to be reduced when the mTOR axis was activated, preventing against myocardial injury during the acute stage of ischemia/reperfusion injury [33]. Importantly, the increased level of LC3-II has been observed as a marker of activated autophagy [34]. Moreover, p62 has been identified as a substrate that can be degraded via the autophagy-lysosomal pathway through LC3 interaction [35]. It has been demonstrated that BDNF is neuroprotective by regulating the autophagy of oxygen-deprived cells through interaction with the Akt/mTOR/ribosomal protein S6 kinase (p70S6 K) axis [36]. The function of mTOR pathway has been shown to negatively regulate autophagy, the activation of which can be partially associated with miR-222 induced autophagy suppression [37].

Furthermore, H9c2 cells transfected with miR-18a inhibitors exhibited an enhanced SOD activity and diminished MDA level, accompanied by up-regulation of BDNF and inactivation of Akt/mTOR axis. This result verified the inhibitory effects of downregulating miR-18a on cellular senescence. Cellular senescence can be defined as cell cycle arrest as a result of DNA damage or other stress, which is featured with enhanced SA-β-gal activity [38]. The present study has revealed that the proportion of SA-β-gal positive cells was decreased in H9c2 cells when treated with miR-18a inhibitors, suggesting that cellular senescence was suppressed when miR-18a was inhibited. Additionally, according to an investigation on senescence of mesenchymal stem cells, more SA-β-gal positive cells were observed in cells when the Akt/mTOR axis was activated, suggesting that activation of the Akt/mTOR axis may be correlated to enhanced cell senescence [39]. The suppressed level of MDA and the activated level of SOD have been proven to be signs of AMI amelioration in a rat model [40]. Researchers have mentioned that BDNF can elevate SOD expression level in hippocampal neurons of rats [41], which is consistent with the findings obtained.

## Conclusions

**Fig. 7:**
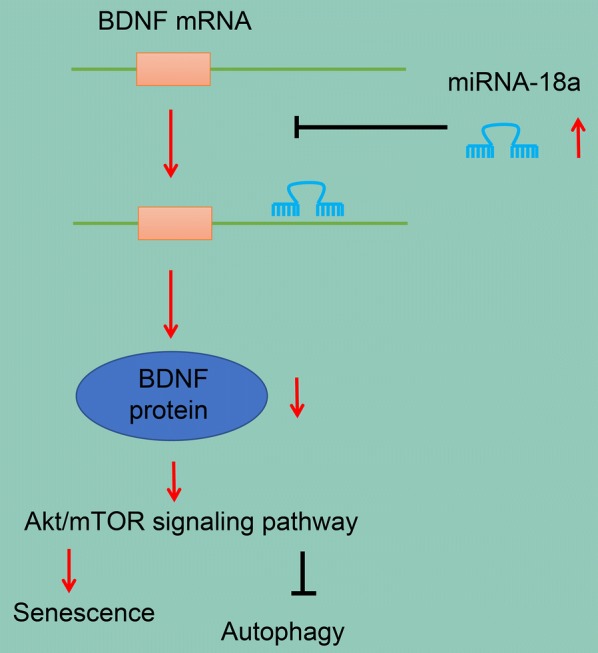
The schematic diagram depicts molecular basis of miR-18a involving in the autophagy, and senescence of cardiomyocytes after AMI via the BDNF-dependently Akt/mTOR axis. In the cardiomyocytes from an AMI rat model, miR-18a expression was up-regulated, which inhibited the expression of BDNF and activated the Akt/mTOR axis, thereby promoting the senescence and inhibiting the autophagy of cardiomyocytes

In conclusion, the study proposes that down-regulation of miR-18a inhibits senescence while promoting autophagy of cardiomyocytes in rats with AMI via upregulating BDNF and inactivating Akt/mTOR axis (Fig. 7). This can be considered as a potential predictive biomarker for the AMI treatment. In this regards, the current study provides an insight into the molecular mechanism of miR-18a in AMI, which may promote the development of miRNA-directed diagnoses and therapy for cardiac disorder in the near future.

## Abbreviations

AMI: acute myocardial infarction
miRNAs: microRNAs
BDNF: brain derived neurotrophic factor
mTOR: mammalian target of rapamycin
SPF: specific pathogen free
ECG: electrocardiogram
LAD: left anterior descending
LAA: left atrial appendage
NC: negative control
TTC: triphenyltetrazolium chloride
RT-qPCR: reverse transcription quantitative polymerase chain reaction
IS: infarct size
MS: myocardium size
OD: optical density
DAB: diaminobenzidine
NCBI: National Center for Biotechnology Information
UTR: untranslated region
SA-β-gal: senescence-associated β-galactosidase
SOD: superoxide dismutase
MDA: malondialdehyde
ANOVA: analysis of variance
